# Association of serum uric acid with visceral, subcutaneous and hepatic fat quantified by magnetic resonance imaging

**DOI:** 10.1038/s41598-020-57459-z

**Published:** 2020-01-16

**Authors:** Susanne Rospleszcz, Ditjon Dermyshi, Katharina Müller-Peltzer, Konstantin Strauch, Fabian Bamberg, Annette Peters

**Affiliations:** 1 Institute of Epidemiology, Helmholtz Zentrum München - German Research Center for Environmental Health, Neuherberg, Germany; 2 Department of Medical Information Processing, Biometry, and Epidemiology, Faculty of Medicine, Ludwig-Maximilians-Universität München, Munich, Germany; 3 Regional Hospital of Mindelheim, Department of Internal Medicine, Mindelheim, Germany; 4 Department of Diagnostic and Interventional Radiology, Medical Center-University of Freiburg, Faculty of Medicine, University of Freiburg, Freiburg, Germany; 5 Institute of Genetic Epidemiology, Helmholtz Zentrum München - German Research Center for Environmental Health, Neuherberg, Germany; 6 Chair of Genetic Epidemiology, Institute for Medical Information Processing, Biometry, and Epidemiology, Faculty of Medicine, Ludwig-Maximilians-Universität München, Munich, Germany; 7 Chair of Epidemiology, Faculty of Medicine, Ludwig-Maximilians-Universität München, Munich, Germany

**Keywords:** Diagnostic markers, Epidemiology

## Abstract

Elevated serum uric acid (SUA) is associated with a variety of medical conditions, such as hypertension, diabetes and obesity. Analyses investigating uric acid and obesity were primarily conducted using anthropometric measures like BMI and waist circumference. However, different adipose tissue depots might be differentially affected in uric acid metabolism. We analyzed the relation of SUA with visceral, subcutaneous and hepatic fat as quantified by Magnetic Resonance Imaging in N = 371 individuals from a cross-sectional sample of a population-based cohort. Associations of SUA and fat depots were calculated by regressions adjusted for potential confounders. We found that SUA was correlated with all fat measures (e.g. Pearson’s r between SUA and hepatic fat: 0.50, 95%-CI: 0.42, 0.57). Associations with visceral and hepatic fat, but not with subcutaneous fat, remained evident after adjustment for anthropometric measures (e.g. visceral fat: β = 0.51 l, 95%-CI: 0.30 l, 0.72 l). In conclusion, these results show how different adipose tissue compartments are affected by SUA to varying degrees, thus emphasizing the different physiological roles of these adipose tissues in uric acid metabolism.

## Introduction

Serum Uric Acid (SUA) originates from the metabolic breakdown of purine nucleotides. When SUA exceeds the normal range of homeostasis, it is deposited in articulations and soft tissues in the form of monosodium urate crystals. Thus, elevated SUA levels (hyperuricemia) are the major etiologic factor for developing gout^1^. However, hyperuricemia has also been linked to several metabolic disorders such as chronic kidney disease^2,3^, fatty liver^4^, metabolic syndrome^5–7^ and its components, including hypertension^8–10^, diabetes^11–13^ and adiposity^14,15^.

Prevalence of adiposity and fatty liver – and thus also the prevalence of related comorbidities – is rising^16,17^. It is therefore important to investigate biomarkers such as SUA that might help to understand pathways and mechanisms of adipose tissue metabolism and examine their potential causal role in the development of adiposity.

However, the investigation of the association of SUA with adiposity and fatty liver is often hampered by insufficient methodology to quantify adipose tissue content. Adiposity is usually determined by anthropometric measures such as waist circumference (WC) and Body Mass Index (BMI); but these crude measures do not give a complete picture of the fat distribution in the body and cannot quantify the amount of metabolically active adipose tissue. Adipose tissue is a heterogeneous entity and different compartments might be affected by SUA in different ways. A recent Mendelian Randomization study found that genetically predicted BMI was associated with the risk of gout, whereas increased waist circumference was not, indicating the differential involvement of adipose tissue in uric acid metabolism^18^. Therefore, accurate and differential quantification of body adipose tissue is needed.

There have already been efforts to use medical imaging such as computed tomography (CT) to determine adipose tissue in the context of its association with SUA^14,15,19^. However, magnetic resonance imaging (MRI) has emerged as the gold standard for the quantification of adipose tissue, due to its high sensitivity and high resolution. Strong evidence of a correlation of SUA with MRI-derived abdominal adipose tissue and hepatic fat in a population-based sample is still lacking.

We therefore aim to analyze the association of SUA as an exposure variable with outcomes of MRI-derived visceral, subcutaneous and hepatic fat fraction (HFF) in a population-based sample.

## Materials and Methods

### Study population

**Figure 1:**
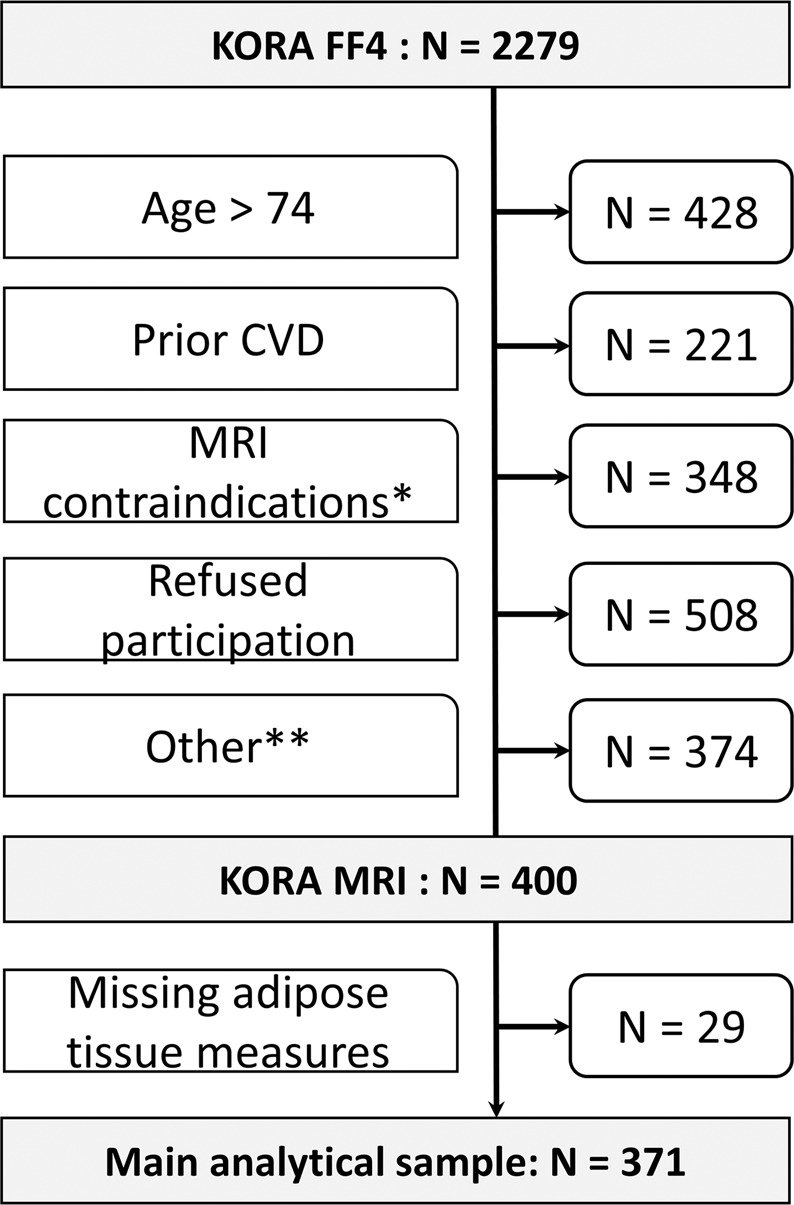
Flowchart of participants. CVD: cardiovascular disease defined as myocardial infarction, stroke or revascularization; * non-removable metal parts in the body, such as pacemakers or stents, renal insufficiency, known gadolinium allergy, claustrophobia, inability to lay down and/or hold breath, pregnancy or breast-feeding; ** unreachable by phone call (n = 39), scheduling problems (n = 8) and not included for matching (n = 327).

The KORA-MRI study includes a cross-sectional sample of 400 participants from a population-based cohort of The Cooperative Health Research in the Augsburg Region (KORA) in Southern Germany. The general design and setup of the KORA studies has been described previously^20^. Briefly, the baseline survey (S4) from 1999/2001 included 4261 participants who were randomly sampled from the city of Augsburg and two adjacent counties. The first follow-up (F4) took place in 2006/2008 and included 3080 of the original participants. The second follow-up (FF4) was conducted in 2013/2014 with 2279 participants. Among the participants of the second follow-up, a whole body MRI was performed on 400 individuals who met the inclusion criteria, as detailed in Fig. 1. The main setup of the KORA-MRI study has been described previously^21^.

### Ethics approval and consent to participate

The KORA FF4 study was approved by the ethics committee of the Bavarian Chamber of Physicians, Munich; the MRI sub-study was approved by the institutional review board of the Ludwig-Maximilians-University Munich. The investigations were carried out in accordance with the Declaration of Helsinki, including written informed consent of all participants.

### Outcome assessment

All MRI scans were done on a 3 Tesla Magnetom Skyra (Siemens Healthineers, Erlangen, Germany), as detailedly described elsewhere^21^. For the assessment of adipose tissue, a volume-interpolated three-dimensional in/opposed-phase VIBE-Dixon sequence was used and adipose tissue was quantified semiautomatically using a Matlab-based (MathWorks Inc., Natick, Massachusetts) inhouse algorithm. Visceral Fat (VAT) was calculated from the femoral head to the diaphragm and subcutaneous fat (SAT) from the femoral head to the cardiac apex. VAT and SAT are summed up to total abdominal adipose tissue. All measurements are indicated in liter (l)^22,23^.

Multi-echo single voxel proton magnetic resonance spectroscopy, based on a high-speed T2-corrected multi-echo technique (HISTO), was used to quantify HFF in the right and left lobe of the liver. The arithmetic mean of left and right lobe HFF was used for further analysis (HFF_HISTO). Additionally, a multiecho Dixon-sequence was utilized to quantify HFF at the level of portal vein (HFF_dixon)^24^. HFF is given in %.

### Exposure and covariate assessment

Venous blood samples were taken without stasis as a part of the standardized examination procedure at the study center. SUA was measured using an enzymatic colorimetric test based on uric acid cleavage by uricase forming allantoin and hydrogen peroxide (Cobas C, Roche). Hyperuricemia was defined as SUA levels >6 mg/dL in women and >7 mg/dL in men^25^. Standard enzymatic and immunonephelometric assays were used to quantify serum cholesterol and albumin levels, respectively.

Weight and height of participants were measured by Seca’s digital scales (Seca GmbH & Co, KG, Hamburg, Germany), determined to the closest 0.1 kg and 0.1 cm, respectively. BMI was computed as weight in kg divided by height in m squared. WC was measured with an inelastic measuring tape between the iliac crest and lower rib margin.

Systolic and diastolic blood pressure were measured with an automated oscillometric device (Omron HEM-705CP) while participants were seated. Three measurements with three-minute intervals in between were obtained and the mean of the second and third measurement was used as the final value.

Participants’ glycemic status was categorized as either type 2 diabetes (established type 2 diabetes or newly detected type 2 diabetes by Oral Glucose Tolerance Test (OGTT) with 2-h glucose ≥200 mg/dL and/or fasting glucose ≥125 mg/dL according to WHO criteria), prediabetes (2-h glucose between 140 and 200 mg/dL and/or fasting glucose between 110 and 125 mg/dL according to WHO criteria) or normoglycemic.

Smoking history, alcohol consumption and medication intake were assessed by self-report during the standardized interview.

Medication was considered antihypertensive if it contained antihypertensive agents according to the most current guidelines of the German Hypertension Association and if individuals were aware of having hypertension. Diuretics, lipid-lowering medication and gout medication were defined according to the Anatomical Therapeutic Chemical (ATC) classification as C03, C10 and M04, respectively.

### Statistical methods

Descriptive characteristics of the study population, stratified by presence of hyperuricemia, are presented as arithmetic mean and SD for continuous covariables and as counts and percentages for categorical covariables. Differences were evaluated by t-test or χ^2^-Test, where applicable.

Correlation between SUA and measures of adipose tissue were assessed graphically by scatterplots and quantitatively by Pearson’s correlation coefficient with respective 95% confidence intervals (CI). Differences in adipose tissue measures according to presence of hyperuricemia were assessed graphically by boxplots and quantitatively by t-test.

To assess the association between SUA and adipose tissue, we used a multiple linear regression model adjusted for age, sex, systolic blood pressure, total serum cholesterol, serum albumin, alcohol consumption, gout medication, diuretic medication, antihypertensive medication, lipid-lowering medication, antidiabetic medication and glycemic status. SUA served as the exposure variable and adipose tissue (VAT, SAT, hepatic fat fraction) as the outcome. Continuous exposure and adjustment variables were standardized (mean = 0, standard deviation = 1) before analysis. The outcome hepatic fat fraction (HFF_dixon and HFF_HISTO) was log-transformed and resulting estimates therefore denote the percent change in mean HFF. Goodness-of-fit of the models was estimated by the R^2^ metric which denotes the percentage of variance in the outcome that is explained by the model. In a second step, the model was additionally adjusted for either BMI or WC. We calculated all models for continuous exposure SUA and dichotomized exposure hyperuricemia; for the whole sample as well as in a sex-stratified fashion. In a sensitivity analysis, all individuals taking anti-gout medication were excluded. In another sensitivity analysis, fasting serum glucose instead of glycemic status was used for adjustment.

R version 3.4.4 was used for all analyses.

## Results

### Characteristics of the study sample

**Figure 2:**
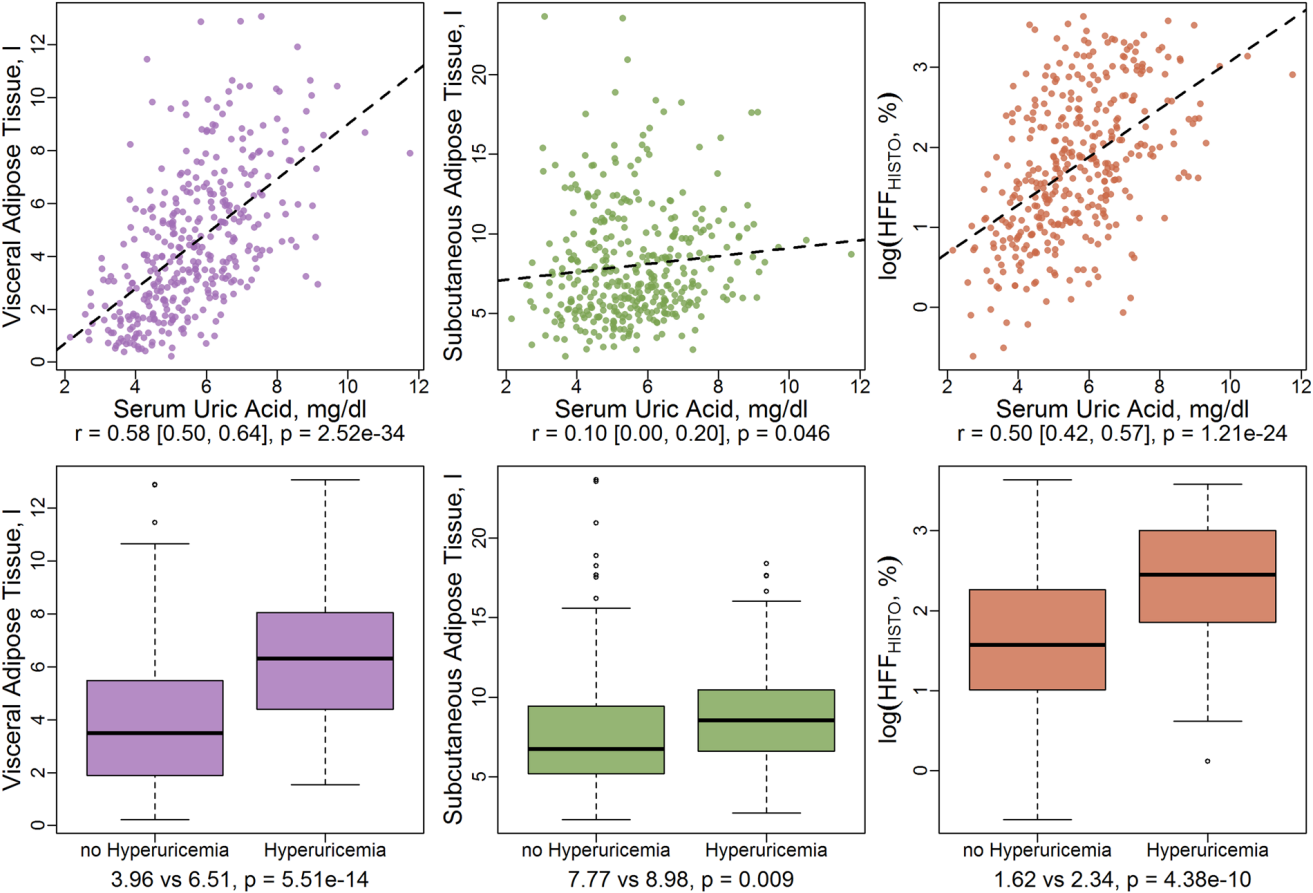
Correlation of serum uric acid and hyperuricemia with MRI-derived adipose tissue compartments.

Baseline characteristics of the study sample, stratified by presence of hyperuricemia, are given in Tables 1 and 2. Sex-stratified results are provided in Supplementary Table S1. The sample comprised 371 middle-aged individuals (mean age 56.1 ± 9.1 years) with a mean SUA level of 5.6 ± 1.5 mg/dL. Prevalence of hyperuricemia was almost 20%, with a higher prevalence in men (26.3%) than in women (10.4%). Participants with hyperuricemia were on average older, had higher BMI and WC, higher blood pressure and generally a more unfavorable risk profile (compare Table 1). The amount of VAT, SAT and HFF was evidently higher in participants with hyperuricemia compared to those without (compare Table 2 and boxplots in Fig. 2). Continuous SUA was correlated to VAT (r = 0.58, p = 2.52E-24) and HFF (r = 0.50, p = 1.21E-24) and to a lesser extent to SAT (r = 0.10, p = 0.04, compare scatterplots in Fig. 2).

**Table 1:** Baseline characteristics of the sample.

	Whole sample	no Hyperuricemia	Hyperuricemia	pvalue
	N = 371	N = 298 (80.3%)	N = 73 (19.7%)	
Age, years	56.1 ± 9.1	55.6 ± 9.1	57.9 ± 8.8	0.054
Men	217 (58.5%)	160 (53.7%)	57 (78.1%)	2.54E-04
BMI, kg/m^2^	27.8 ± 4.7	27.3 ± 4.6	29.8 ± 4.3	4.19E-05
Waist circumference, cm	97.9 ± 13.7	95.9 ± 13.6	106.1 ± 10.7	4.79E-09
Systolic Blood Pressure, mmHg	120.7 ± 16.5	118.8 ± 15.6	128.1 ± 18.1	1.31E-05
Diastolic Blood Pressure, mmHg	75.4 ± 9.9	74.9 ± 9.6	77.4 ± 10.7	0.052
Albumin, mg/dL	4.3 ± 0.3	4.3 ± 0.3	4.4 ± 0.3	0.033
Total cholesterol, mg/dL	217.6 ± 36.4	216.9 ± 35.4	220.3 ± 40.4	0.482
HDL cholesterol, mg/dL	61.9 ± 17.8	63.4 ± 17.6	55.8 ± 17.2	9.86E-04
LDL cholesterol, mg/dL	139.3 ± 32.9	139.1 ± 32.6	140.3 ± 34.1	0.788
Triglycerides, mg/dL	131.7 ± 86.1	119.9 ± 69.4	180.1 ± 123.4	4.98E-08
Creatinine, mg/dL	0.9 ± 0.2	0.9 ± 0.2	1.0 ± 0.1	5.44E-07
Uric acid, mg/dL	5.6 ± 1.5	5.1 ± 1.1	7.7 ± 1.0	4.12E-57
Antihypertensive medication	90 (24.3%)	66 (22.1%)	24 (32.9%)	0.078
Lipid-lowering medication	39 (10.5%)	32 (10.7%)	7 (9.6%)	0.941
Gout medication	9 (2.4%)	8 (2.7%)	1 (1.4%)	1
Diuretic medication	45 (12.1%)	28 (9.4%)	17 (23.3%)	0.002
Antidiabetic medication	29 (7.8%)	22 (7.4%)	7 (9.6%)	0.699
**Smoking**				
never-smoker	134 (36.1%)	107 (35.9%)	27 (37.0%)	0.060
ex-smoker	161 (43.4%)	123 (41.3%)	38 (52.1%)	
smoker	76 (20.5%)	68 (22.8%)	8 (11.0%)	
Alcohol consumption, g/day	18.9 ± 24.2	17.0 ± 23.0	26.4 ± 27.3	0.003
thereof beer	11.6 ± 18.9	9.3 ± 16.4	20.7 ± 24.8	2.75E-06
thereof wine	6.8 ± 14.1	7.2 ± 14.3	5.1 ± 12.9	0.246
thereof spirits	0.5 ± 1.6	0.5 ± 1.6	0.6 ± 1.4	0.642
Fasting serum glucose, mg/dL	104.0 ± 22.7	103.4 ± 24.2	106.6 ± 15.1	0.271
**Glycemic Status**				
normoglycemic	231 (62.3%)	195 (65.4%)	36 (49.3%)	0.039
prediabetes	90 (24.3%)	66 (22.1%)	24 (32.9%)	
diabetes	50 (13.5%)	37 (12.4%)	13 (17.8%)	

Continuous variables are presented as mean ± standard deviation with p-values from t-test. Categorical variables are presented as counts and percentage with p-values from Wilcoxon rank-sum test. Hyperuricemia was defined as serum uric acid levels >6 mg/dL in women and >7 mg/dL in men. P-values are exploratory and not corrected for multiple testing.

**Table 2:** Measures of adipose tissue, as derived by MRI.

	Whole sample	no Hyperuricemia	Hyperuricemia	pvalue
	N = 371	N = 298 (80.3%)	N = 73 (19.7%)	
Total abdominal adipose tissue, l	12.5 ± 5.3	11.7 ± 5.2	15.5 ± 4.7	3.68E-08
Visceral adipose tissue, l	4.5 ± 2.7	4.0 ± 2.5	6.5 ± 2.5	5.51E-14
Subcutaneous adipose tissue, l	8.0 ± 3.6	7.8 ± 3.6	9.0 ± 3.3	0.009
HFF_HISTO, %	8.6 ± 7.8	7.4 ± 7.2	13.3 ± 8.5	5.52E-09
HFF_right lobe, %	9.4 ± 8.5	8.1 ± 7.6	14.8 ± 9.9	6.52E-10
HFF_left lobe, %	7.8 ± 7.6	6.8 ± 7.2	11.8 ± 8.0	3.15E-07
log(HFF_HISTO, %)	1.8 ± 0.9	1.6 ± 0.9	2.3 ± 0.8	4.38E-10
HFF_dixon, %	8.3 ± 8.3	7.1 ± 7.7	13.0 ± 9.0	2.62E-08
log(HFF_dixon, %)	1.7 ± 0.9	1.5 ± 0.9	2.3 ± 0.8	3.28E-10

Data are presented as arithmetic means and standard deviations. HFF_HISTO: hepatic fat fraction as measured by high-speed T2-corrected multi-echo technique (HISTO). HFF_dixon: hepatic fat fraction, as measured by multiecho Dixon-sequence. Hyperuricemia was defined as serum uric acid levels >6 mg/dL in women and >7 mg/dL in men. P-values are exploratory and not corrected for multiple testing.

### Cross-sectional association of SUA/hyperuricemia and adipose tissue

Results of the adjusted linear regression models describing the cross-sectional association of SUA or hyperuricemia with the different adipose tissue outcomes are presented in Table 3. A change of one standard deviation of SUA was associated with an increase of 0.82 l in VAT. The association was attenuated after adjustment for BMI and even further attenuated after adjustment for WC, but was still highly evident. Effect size estimates for the dichotomous variable hyperuricemia were even more pronounced.

**Table 3:** Association of SUA and Hyperuricemia with MRI-derived adipose tissue content.

	VAT				SAT				HFF_HISTO				HFF_dixon			
	β	95%-CI	p-value	R^2^	β	95%-CI	p-value	R^2^	Estimate	95%-CI	p-value	R^2^	Estimate	95%-CI	p-value	R^2^
Exposure SUA																
age + sex adjusted	1.03	[0.77, 1.29]	4.8E-14	0.43	1.19	[0.76, 1.62]	9.6E-08	0.10	1.46	[1.32, 1.62]	1.3E-13	0.28	1.48	[1.34, 1.63]	3.8E-13	0.27
men	0.84	[0.53, 1.15]	2.79E-07	0.18	0.89	[0.48, 1.31]	3.34E-05	0.08	1.30	[1.16, 1.45]	4.47E-06	0.12	1.28	[1.15, 1.43]	1.20E-05	0.11
women	0.86	[0.60, 1.11]	1.08E-09	0.36	1.06	[0.44, 1.68]	8.69E-04	0.08	1.49	[1.32, 1.67]	1.79E-10	0.34	1.54	[1.36, 1.73]	1.68E-10	0.32
fully adjusted	0.82	[0.58, 1.07]	2.20E-10	0.56	0.81	[0.39, 1.24]	1.87E-04	0.27	1.32	[1.20, 1.45]	1.07E-08	0.44	1.31	[1.20, 1.45]	4.56E-08	0.45
men	0.66	[0.36, 0.96]	2.19E-05	0.34	0.65	[0.25, 1.04]	0.002	0.26	1.19	[1.07, 1.31]	6.70E-04	0.38	1.16	[1.05, 1.28]	0.003	0.38
women	0.55	[0.30, 0.79]	2.18E-05	0.54	0.5	[−0.11, 1.12]	0.110	0.26	1.36	[1.2, 1.54]	1.72E-06	0.40	1.38	[1.21, 1.57]	1.99E-06	0.42
fully adjusted + WC	0.39	[0.20, 0.58]	8.06E-05	0.76	−0.15	[−0.37, 0.08]	0.198	0.81	1.19	[1.09, 1.30]	1.22E-04	0.54	1.17	[1.07, 1.28]	3.96E-04	0.55
men	0.23	[0.01, 0.45]	0.038	0.67	−0.04	[−0.25, 0.18]	0.725	0.80	1.08	[0.99, 1.19]	0.082	0.51	1.07	[0.97, 1.17]	0.170	0.49
women	0.33	[0.15, 0.51]	4.83E-04	0.76	−0.18	[−0.50, 0.14]	0.272	0.81	1.27	[1.14, 1.43]	4.00E-05	0.49	1.27	[1.14, 1.43]	5.31E-05	0.54
fully adjusted + BMI	0.51	[0.30, 0.72]	2.08E-06	0.70	0	[−0.21, 0.22]	0.976	0.82	1.23	[1.13, 1.35]	8.28E-06	0.51	1.22	[1.12, 1.34]	3.06E-05	0.52
men	0.34	[0.09, 0.58]	0.007	0.59	0.05	[−0.16, 0.27]	0.633	0.80	1.11	[1.01, 1.21]	0.029	0.47	1.09	[0.99, 1.2]	0.069	0.45
women	0.38	[0.19, 0.56]	1.13E-04	0.74	−0.07	[−0.36, 0.23]	0.659	0.84	1.31	[1.16, 1.46]	1.41E-05	0.46	1.31	[1.16, 1.48]	1.87E-05	0.51
Exposure Hyperuricemia																
age + sex adjusted	1.76	[1.22, 2.31]	7.53E-10	0.40	1.62	[0.71, 2.53]	5.33E-04	0.22	1.72	[1.39, 2.12]	7.28E-07	0.22	1.77	[1.42, 2.20]	4.92E-07	0.21
men	1.55	[0.83, 2.27]	3.13E-05	0.15	1.30	[0.34, 2.26]	8.23E-03	0.03	1.55	[1.21, 1.99]	5.61E-04	0.08	1.60	[1.25, 2.05]	3.25E-04	0.08
women	2.41	[1.54, 3.27]	1.74E-07	0.31	2.39	[0.35, 4.42]	2.17E-02	0.04	2.20	[1.46, 3.29]	1.67E-04	0.21	2.32	[1.49, 3.56]	2.27E-04	0.19
fully adjusted	1.48	[0.99, 1.98]	9.50E-09	0.55	1.10	[0.25, 1.94]	0.011	0.25	1.48	[1.22, 1.79]	7.59E-05	0.41	1.49	[1.22, 1.80]	6.62E-05	0.43
men	1.33	[0.68, 1.98]	7.82E-05	0.34	1.02	[0.15, 1.89]	0.022	0.24	1.39	[1.12, 1.72]	0.003	0.37	1.39	[1.12, 1.72]	0.003	0.38
women	1.75	[0.95, 2.55]	2.82E-05	0.54	1.09	[−0.93, 3.10]	0.288	0.25	1.73	[1.14, 2.64]	0.010	0.33	1.70	[1.09, 2.64]	0.019	0.34
fully adjusted + WC	0.87	[0.50, 1.24]	5.49E-06	0.76	−0.24	[−0.67, 0.20]	0.284	0.81	1.26	[1.06, 1.49]	9.01E-03	0.53	1.27	[1.06, 1.51]	0.008	0.54
men	0.62	[0.16, 1.08]	0.009	0.68	−0.11	[−0.56, 0.35]	0.638	0.80	1.19	[0.98, 1.43]	0.075	0.51	1.21	[0.99, 1.48]	0.060	0.49
women	1.32	[0.75, 1.88]	9.94E-06	0.77	−0.24	[−1.27, 0.78]	0.643	0.81	1.52	[1.03, 2.23]	0.033	0.45	1.43	[0.97, 2.12]	0.067	0.49
fully adjusted + BMI	1.06	[0.66, 1.47]	3.58E-07	0.71	0.05	[−0.37, 0.46]	0.827	0.82	1.34	[1.12, 1.58]	1.72E-03	0.49	1.34	[1.12, 1.62]	0.002	0.51
men	0.89	[0.37, 1.40]	7.74E-04	0.60	0.22	[−0.24, 0.67]	0.352	0.80	1.26	[1.04, 1.54]	0.021	0.47	1.27	[1.04, 1.55]	0.018	0.46
women	1.24	[0.63, 1.84]	9.14E-05	0.74	−0.64	[−1.59, 0.30]	0.182	0.84	1.52	[1.02, 2.27]	0.040	0.40	1.42	[0.94, 2.14]	0.089	0.45

Results from a linear regression model with outcome adipose tissue for the whole study sample, as well as sex-stratified. Exposure was either the continuous variable SUA or the dichotomous variable Hyperuricemia, defined as serum uric acid levels >6 mg/dL in women and >7 mg/dL in men. The fully adjusted model was adjusted for age, sex, systolic blood pressure, total serum cholesterol, serum albumin, alcohol consumption, gout medication, diuretic medication, antihypertensive medication, lipid-lowering medication, antidiabetic medication and glycemic status. Outcome hepatic fat fraction (HFF) was log-transformed and resulting estimates therefore denote the percent change in mean HFF. Continuous exposure and adjustment variables were standardized (mean = 0, standard deviation = 1) before analysis; therefore estimates for models with exposure SUA denote the change in adipose tissue per one standard deviation of SUA. Note that for sex-stratified models, standardization was also performed in a stratified fashion. R^2^ denotes the percentage of variance in the outcome that is explained by the model. P-values are exploratory and not corrected for multiple testing. VAT: visceral adipose tissue; SAT: subcutaneous adipose tissue; HFF_HISTO: hepatic fat fraction as measured by high-speed T2-corrected multi-echo technique (HISTO); HFF_dixon: hepatic fat fraction, as measured by multiecho Dixon-sequence.

A change of one standard deviation of SUA was associated with an increase of 0.81 l in SAT, but this model could only explain 27% of the variation in SAT. After adjustment for BMI or WC, more than 80% of the variation in SAT could be explained, but the association of SUA was attenuated (compare Table 3).

Results for HFF were similar for the average HFF of the right and left liver lobe (HFF_HISTO) and HFF measured at the level of the portal vein (HFF_dixon). A change in one standard deviation of SUA was associated with an increase of 32% in HFF_HISTO and an increase of 31% in HFF_dixon. Comparable to the results for VAT, the association of SUA with HFF was attenuated after adjustment for BMI or WC, but was still evident; and the effect sizes for the dichotomous variable hyperuricemia were larger in size.

In a sensitivity analysis excluding all participants taking anti-gout medication, similar results were obtained (Supplementary Table S2). Furthermore, in another sensitivity analysis adjusting for serum fasting glucose instead of glycemic status, similar results were obtained (Supplementary Table S3).

Results for the male and female subsample were similar. However, the association of SUA and hyperuricemia with HFF was more pronounced in women.

## Discussion

In this population-based sample, SUA and hyperuricemia were strongly related to increased storage of VAT and hepatic fat, independently of additional confounders and independently of anthropometric measures of overall adiposity. In contrast, the association of SUA and SAT was not independent of anthropometric measures.

The relation between SUA and anthropometric measures of overall adiposity is well established. Several studies found cross-sectional associations between BMI or WC with SUA or hyperuricemia^26–28^. Moreover, SUA levels have predictive ability with respect to weight gain. In normotensive, non-obese men, baseline SUA predicted 5-year changes in BMI^29^. A more recent study found that hyperuricemia in individuals without any other comorbidities predicted an increased 5-year risk of developing obesity, independent of other confounders^30^.

Without the appropriate methodology, VAT and SAT cannot be quantified separately. Therefore, analyses of their specific effects are constrained to studies using medical imaging; however those studies have mainly focused on total abdominal fat without drawing any distinction of VAT and SAT. In this regard, our results are in line with previous studies. In a cross-sectional study of 801 healthy Japanese men, Yamada *et al*. found an independent association between both hepatic and visceral fat tissue quantified by CT with hyperuricemia^19^. Another study using CT for quantification of visceral adiposity found that elevated SUA levels were associated with visceral fat accumulation^31^. Yet to our knowledge epidemiological studies assessing the relationship between subcutaneous adipose tissue and SUA are scarce.

Adipose tissue is a central endocrine organ and both VAT and SAT have key metabolic functions in energy homeostasis and glucose regulation. However, they have different cellular properties and exhibit major functional differences. Recent preliminary ontogenetic studies suggest that VAT and SAT cells originate from different embryonic mesodermal regions^32^.

The role of SUA on a cellular level is not quite clear. Mouse models have shown that adipose tissues can produce and secrete uric acid and secretion is enhanced in obesity^33^. Uric acid is also involved in the production of key pro-inflammatory adipokines in adipose tissue^34^. Especially VAT secretes pro-inflammatory adipocytokines and cytokine‐like factors such as tumor necrosis factor α and interleukin-6, thereby contributing to chronic low-grade inflammation. This might be mainly due to accelerated hypertrophic lipogenesis without appropriate angiogenesis, thus resulting in hypoxia, apoptotic and necrotic adipocytes, inflammation and an overbalance of unfavorably polarized M1-macrophages^35^. Our results demonstrate a strong cross-sectional association of SUA with VAT, beyond BMI and WC.

SAT, on the other hand, has a higher secretion of adiponectin, which has anti-inflammatory and insulin-sensitizing properties. Uric acid inhibits adiponectin production in adipocytes^34,36^ Adiponectin levels are negatively correlated with uric acid^37^ and adiponectin production is lower in obese individuals. In our analysis, we found higher amounts of SAT in individuals with hyperuricemia, but this association was attenuated after adjustment for anthropometric measures.

In the context of hepatic fat, cross-sectional associations of elevated SUA and NAFLD have been reported from smaller clinical and larger epidemiological studies^38–41^. Moreover, a predictive role of SUA has been suggested. In a 5-year retrospective cohort study of 4954 healthy Korean participants, whose intrahepatic fat was quantified using abdominal ultrasonography, Lee *et al*. found an association between hyperuricemia and an increased risk of developing NAFLD^42^. Jensen *et al*. used data from 8025 participants of a retrospective Japanese cohort study and found that accelerated SUA increase over a period of five years was associated with a higher risk of developing NAFLD from a healthy liver, independently of other metabolic confounders^43^.

Anatomy suggests a close interrelation of VAT and hepatic fat, as inflammation-enhancing adipokines and free fatty acids that are secreted from VAT can be directly deposited in the liver through the portal vein. In line with this, our results relating SUA to hepatic fat were similar to those relating SUA to VAT, as both showed strong cross-sectional associations which pertained after adjustment for BMI or WC. Several pathways potentially link hyperuricemia to hepatic lipogenesis: First, increased intra-cellular uric acid levels affect hepatocyte mitochondrial function. Thus, they promote an increased accumulation of reactive oxygen species and induce stress on the endoplasmatic reticulum which results in lipogenesis^44,45^. Second, increased uric acid levels upregulate fructokinase, an enzyme responsible for dietary fructose metabolism. Overexpression of fructokinase modulates the lipogenic effects of fructose by inducing increased triglyceride accumulation in hepatocytes^46^. Third, uric acid leads to downregulation of adenosine monophosphate activated kinase (AMPK). Lower expression of AMPK leads to reduced lipolysis, i.e. lower depletion of hepatocytes, lower fat oxidation and higher lipogenesis^47^. However, the directionality of this effect is not straightforward, as fatty liver has also been found to be a predictor of incident hyperuricemia^48,49^.

In sex-stratified analyses, we found similar tendencies for men and women. However, estimates of the association of SUA with hepatic fat were substantially higher in women. This is in line with two other population-based studies that found stronger associations of uric acid with NAFLD in women compared to men^50,51^. However, as other studies report stronger associations in men^52,53^, findings remain inconclusive. Genetic and hormonal sex differences in the development and pathophysiology of NAFLD have been established (recently reviewed by Lonardo *et al*.^54^) but the exact mechanism that would influence the differential association of SUA to hepatic fat is not yet clear.

Our study only comprises cross-sectional observational data and thus cannot establish the direction of the relation between SUA and adipose tissue nor its causality. Further research on larger, longitudinal cohort studies is needed to investigate the issue. Another option is the use of Mendelian Randomization analyses to investigate causality. In this respect, a recent meta-GWAS of serum urate identified 147 previously unknown SNPs which could be used to build an informative genetic instrument for such a Mendelian Randomization analysis^55^. On the other hand, data on the association of these SNPs with VAT, SAT and hepatic fat would be needed as well. Although GWAS of VAT, SAT and non-alcoholic fatty liver disease (NAFLD) have been conducted^56–58^, genome-wide summary statistics - i.e. results for every analyzed SNP and not only the significant ones - are not publicly available. In theory, these associations could also be estimated from our sample at hand, but given the limited size of the study sample, and given the fact that genetic effects are usually extremely small, such an estimation is severely statistically underpowered. Therefore, these analyses constitute interesting future research, when more GWAS data and increased sample sizes become available.

Besides these limitations, a major strength of our study is the use of a population-based sample from a well-characterized, prospective cohort study and the use of MRI, which allows a precise quantification of VAT, SAT and hepatic fat. Furthermore, we can rule out confounding by cardiovascular disease or renal impairment, as these were global exclusion criteria for the study sample.

In conclusion, our findings show how VAT, SAT and hepatic fat are differentially affected by serum uric acid, thus highlighting the different physiological roles of these fat compartments in uric acid metabolism.

## Supplementary information

Supplementary material.

## Data Availability

Informed consent given by KORA study participants does not cover data posting in public databases. However, data are available upon request from KORA-gen (http://epi.helmholtz-muenchen.de/kora-gen/) by means of a project agreement. Requests should be sent to kora.passt@helmholtz-muenchen.de and are subject to approval by the KORA Board.
