# Supplementary material for: Association of serum uric acid with visceral, subcutaneous and hepatic fat quantified by magnetic resonance imaging
Source: Sci Rep. 2020 Jan 16;10:442. doi: 10.1038/s41598-020-57459-z (PMC6965096; doi:10.1038/s41598-020-57459-z)
Supplement: Supplementary file 1 — Supplementary material. [file 41598_2020_57459_MOESM1_ESM.pdf]

Supplementary material to

Association of serum uric acid with visceral, subcutaneous and hepatic fat quantified by magnetic resonance imaging

Susanne Rospleszcz[1], Ditjon Dermyshi[1,2,3], Katharina Müller-Peltzer[4], Konstantin Strauch[5,6], Fabian Bamberg[4], Annette Peters[1,7]

[1] Institute of Epidemiology, Helmholtz Zentrum München - German Research Center for Environmental Health, Neuherberg, Germany
[2] Department of Medical Information Processing, Biometry, and Epidemiology, Faculty of Medicine, Ludwig-Maximilians-Universität München, Munich, Germany
[3] Regional Hospital of Mindelheim, Department of Internal Medicine, Mindelheim, Germany
[4] Department of Diagnostic and Interventional Radiology, Medical Center-University of Freiburg, Faculty of Medicine, University of Freiburg, Freiburg, Germany
[5] Institute of Genetic Epidemiology, Helmholtz Zentrum München - German Research Center for Environmental Health, Neuherberg, Germany
[6] Chair of Genetic Epidemiology, Institute for Medical Information Processing, Biometry, and Epidemiology, Faculty of Medicine, Ludwig-Maximilians-Universität München, Munich, Germany
[7] Chair of Epidemiology, Faculty of Medicine, Ludwig-Maximilians-Universität München, Munich, Germany

Supplementary Table S1: Characteristics of the study population, sex-stratified

Supplementary Table S1A: Characteristics of the male study sample

	All male individuals<br>N = 217	no Hyperuricemia<br>N = 160 (73.7%)	Hyperuricemia<br>N = 57 (26.3%)	pvalue
Age, years	56.0 ± 9.3	55.7 ± 9.4	56.9 ± 9.0	0.407
BMI, kg/m^2	28.3 ± 4.1	27.9 ± 4.2	29.4 ± 3.7	0.014
Waist circumference, cm	102.9 ± 11.6	101.4 ± 11.8	107.2 ± 9.9	0.001
Systolic Blood Pressure, mmHg	126.1 ± 16.0	124.8 ± 15.6	129.7 ± 16.7	0.050
Distolic Blood Pressure, mmHg	77.7 ± 10.2	77.4 ± 10.0	78.5 ± 10.8	0.492
Albumin, mg/dL	4.4 ± 0.3	4.4 ± 0.3	4.4 ± 0.3	0.186
Total cholesterol, mg/dL	216.7 ± 37.8	215.0 ± 35.6	221.5 ± 43.6	0.267
HDL cholesterol, mg/dL	55.7 ± 15.0	56.7 ± 13.9	53.0 ± 17.6	0.110
LDL cholesterol, mg/dL	141.4 ± 33.6	141.1 ± 32.5	142.3 ± 36.6	0.814
Triglycerides, mg/dL	153.6 ± 101.2	138.6 ± 82.0	195.6 ± 134.0	2.22E-04
Creatinine, mg/dL	1.0 ± 0.1	1.0 ± 0.1	1.0 ± 0.1	0.086
Uric Acid, mg/dL	6.3 ± 1.3	5.7 ± 0.8	8.0 ± 0.9	5.13E-43
Antihypertensive medication	50 (23.0%)	34 (21.2%)	16 (28.1%)	0.386
Lipid-lowering medication	23 (10.6%)	19 (11.9%)	4 (7.0%)	0.452
Gout medication	9 (4.1%)	8 (5.0%)	1 (1.8%)	0.451
Diuretic medication	24 (11.1%)	15 (9.4%)	9 (15.8%)	0.280
Antidiabetic medication	19 (8.8%)	14 (8.8%)	5 (8.8%)	1
Smoking				
never-smoker	72 (33.2%)	52 (32.5%)	20 (35.1%)	0.056
ex-smoker	103 (47.5%)	71 (44.4%)	32 (56.1%)	
smoker	42 (19.4%)	37 (23.1%)	5 (8.8%)	
Alcohol consumption, g/d	26.1 ± 27.1	25.0 ± 27.3	29.4 ± 26.5	0.292
thereof beer	18.0 ± 22.1	15.5 ± 20.0	25.1 ± 26.1	0.004
thereof wine	7.5 ± 15.3	8.8 ± 17.2	3.6 ± 6.9	0.028
thereof spirits	0.6 ± 1.9	0.6 ± 2.0	0.6 ± 1.5	0.873
Fasting serum glucose, mg/dL	107.9 ± 25.5	108.5 ± 28.4	106.4 ± 14.5	0.593
Glycemic Status				
normoglycemic	120 (55.3%)	91 (56.9%)	29 (50.9%)	0.663

prediabetes	59 (27.2%)	41 (25.6%)	18 (31.6%)	
diabetes	38 (17.5%)	28 (17.5%)	10 (17.5%)	
Total abdominal adipose tissue, l	13.0 ± 5.2	12.3 ± 5.2	15.2 ± 4.4	2.52E-04
Visceral adipose tissue, l	5.6 ± 2.6	5.2 ± 2.5	6.8 ± 2.3	2.30E-05
Subcutaneous adipose tissue, l	7.4 ± 3.2	7.1 ± 3.3	8.3 ± 2.7	0.010
HFF_HISTO, %	10.5 ± 8.5	9.4 ± 8.2	13.5 ± 8.5	0.002
HFF_right lobe, %	11.3 ± 9.1	10.0 ± 8.4	15.0 ± 10.0	3.64E-04
HFF_left lobe, %	9.6 ± 8.4	8.8 ± 8.4	11.9 ± 8.0	0.014
log(HFF_HISTO, %)	2.0 ± 0.9	1.9 ± 0.9	2.4 ± 0.8	3.84E-04
HFF_dixon, %	9.9 ± 8.4	8.7 ± 8.0	13.1 ± 8.9	0.001
log(HFF_dixon, %)	1.9 ± 0.9	1.8 ± 0.8	2.3 ± 0.8	2.21E-04

Supplementary Table S1B: Characteristics of the female study sample

	All female individuals<br>N = 154	no Hyperuricemia<br>N = 138 (89.6%)	Hyperuricemia<br>N = 16 (10.4%)	pvalue
Age, years	56.1 ± 8.9	55.5 ± 8.9	61.5 ± 7.2	0.010
BMI, kg/m^2	27.2 ± 5.3	26.8 ± 5.0	31.2 ± 6.1	0.001
Waist circumference, cm	90.8 ± 13.4	89.5 ± 12.9	102.2 ± 12.9	2.61E-04
Systolic Blood Pressure, mmHg	113.0 ± 14.0	111.9 ± 12.3	122.7 ± 22.1	0.003
Distolic Blood Pressure, mmHg	72.0 ± 8.3	71.9 ± 8.1	73.3 ± 9.8	0.529
Albumin, mg/dL	4.3 ± 0.3	4.3 ± 0.3	4.3 ± 0.3	0.756
Total cholesterol, mg/dL	218.8 ± 34.2	219.2 ± 35.1	215.9 ± 26.1	0.717
HDL cholesterol, mg/dL	70.6 ± 17.8	71.2 ± 18.3	65.7 ± 11.6	0.249
LDL cholesterol, mg/dL	136.4 ± 31.8	136.8 ± 32.7	133.0 ± 22.6	0.651
Triglycerides, mg/dL	100.9 ± 42.7	98.1 ± 41.9	124.8 ± 43.2	0.018
Creatinine, mg/dL	0.8 ± 0.1	0.8 ± 0.1	0.9 ± 0.1	8.24E-05
Uric Acid, mg/dL	4.6 ± 1.1	4.3 ± 0.8	6.8 ± 0.8	8.54E-23
Antihypertensive medication	40 (26.0%)	32 (23.2%)	8 (50.0%)	0.044
Lipid-lowering medication	16 (10.4%)	13 (9.4%)	3 (18.8%)	0.221
Gout medication	0 (0.0%)	NA	NA	
Diuretic medication	21 (13.6%)	13 (9.4%)	8 (50.0%)	4.26E-05

Antidiabetic medication	10 (6.5%)	8 (5.8%)	2 (12.5%)	0.278
Smoking				
never-smoker	62 (40.3%)	55 (39.9%)	7 (43.8%)	1
ex-smoker	58 (37.7%)	52 (37.7%)	6 (37.5%)	
smoker	34 (22.1%)	31 (22.5%)	3 (18.8%)	
Alcohol consumption, g/d	8.7 ± 14.0	7.8 ± 11.2	15.9 ± 28.2	0.029
thereof beer	2.5 ± 5.4	2.2 ± 4.7	5.1 ± 9.5	0.041
thereof wine	5.9 ± 12.0	5.4 ± 9.7	10.4 ± 24.2	0.117
thereof spirits	0.3 ± 0.9	0.3 ± 0.9	0.4 ± 1.2	0.444
Fasting serum glucose, mg/dL	98.5 ± 16.8	97.4 ± 16.4	107.6 ± 17.7	0.021
Glycemic Status				
normoglycemic	111 (72.1%)	104 (75.4%)	7 (43.8%)	0.014
prediabetes	31 (20.1%)	25 (18.1%)	6 (37.5%)	
diabetes	12 (7.8%)	9 (6.5%)	3 (18.8%)	
Total abdominal adipose tissue, l	11.7 ± 5.4	11.1 ± 5.1	16.7 ± 5.5	7.45E-05
Visceral adipose tissue, l	2.8 ± 2.0	2.5 ± 1.6	5.4 ± 2.8	5.4E-09
Subcutaneous adipose tissue, l	8.8 ± 3.9	8.6 ± 3.8	11.2 ± 4.0	0.009
HFF_HISTO, %	6.0 ± 6.0	5.2 ± 5.0	12.7 ± 8.9	7.51E-07
HFF_right lobe, %	6.7 ± 6.7	5.8 ± 5.7	14.0 ± 9.6	1.52E-06
HFF_left lobe, %	5.3 ± 5.4	4.6 ± 4.5	11.4 ± 8.4	8.41E-07
log(HFF_HISTO, %)	1.4 ± 0.9	1.3 ± 0.8	2.3 ± 0.8	8.73E-06
HFF_dixon, %	6.0 ± 7.5	5.2 ± 6.9	12.8 ± 9.7	9.38E-05
log(HFF_dixon, %)	1.3 ± 0.9	1.2 ± 0.9	2.2 ± 0.9	1.46E-05

Continuous variables are presented as mean ± standard deviation with p-values from t-test. Categorical variables are presented as counts and percentage with p-values from Wilcoxon rank-sum test. Hyperuricemia was defined as serum uric acid levels >6 mg/dL in women and >7 mg/dL in men. P-values are exploratory and not corrected for multiple testing.

Supplementary Table S2: Sensitivity analysis: Association of SUA and Hyperuricemia with MRI-derived adipose tissue content after exclusion of n = 9 individuals taking anti-gout medication

	**VAT**				**SAT**				**HFF_HISTO**				**HFF_dixon**			
	β	95%-CI	p-value	R^2	β	95%-CI	p-value	R^2	Est.	95%-CI	p-value	R^2	Est.	95%-CI	p-value	R^2
Exposure SUA																
Age+sex adjusted	1.13	[0.88, 1.38]	4.71E-17	0.45	1.30	[0.87, 1.73]	6.54E-09	0.12	1.52	[1.38, 1.67]	5.98E-16	0.30	1.52	[1.38, 1.68]	5.66E-15	0.28
fully adjusted	0.86	[0.62, 1.11]	2.04E-11	0.55	0.90	[0.48, 1.32]	2.86E-05	0.29	1.35	[1.23, 1.48]	1.00E-09	0.43	1.34	[1.22, 1.48]	7.05E-09	0.44
fully adjusted + WC	0.42	[0.23, 0.62]	2.52E-05	0.74	-0.09	[-0.32, 0.14]	0.447	0.81	1.21	[1.11, 1.32]	3.49E-05	0.54	1.20	[1.09, 1.31]	1.64E-04	0.54
fully adjusted + BMI	0.55	[0.34, 0.76]	4.19E-07	0.69	0.07	[-0.15, 0.29]	0.523	0.82	1.26	[1.14, 1.38]	1.61E-06	0.50	1.23	[1.13, 1.36]	8.97E-06	0.51
Exposure Hyperuricemia																
Age+sex adjusted	1.89	[1.35, 2.42]	1.97E-11	0.41	1.77	[0.86, 2.68]	1.46E-04	0.08	1.79	[1.45, 2.20]	9.19E-08	0.22	1.84	[1.48, 2.29]	9.85E-08	0.21
fully adjusted	1.51	[1.02, 2.00]	3.44E-09	0.54	1.18	[0.34, 2.02]	0.006	0.27	1.49	[1.23, 1.82]	3.89E-05	0.40	1.51	[1.25, 1.84]	4.33E-05	0.41
fully adjusted + WC	0.89	[0.52, 1.26]	3.44E-06	0.74	-0.17	[-0.61, 0.26]	0.433	0.81	1.27	[1.07, 1.51]	0.007	0.52	1.27	[1.07, 1.52]	0.007	0.53
fully adjusted + BMI	1.09	[0.69, 1.50]	1.92E-07	0.69	0.11	[-0.31, 0.53]	0.601	0.82	1.35	[1.13, 1.62]	0.001	0.48	1.35	[1.13, 1.63]	0.001	0.50

Results from a linear regression model with outcome adipose tissue. Exposure was either the continuous variable SUA or the dichotomous variable Hyperuricemia, defined as serum uric acid levels >6 mg/dL in women and >7 mg/dL in men. The fully adjusted model was adjusted for age, sex, systolic blood pressure, total serum cholesterol, serum albumin, alcohol consumption, diuretic medication and glycemic status. Continuous exposure and adjustment variables were standardized (mean = 0, standard deviation = 1) before analysis. Outcome hepatic fat fraction (HFF) was log-transformed and resulting estimates therefore denote the percent change in mean HFF. Continuous exposure and adjustment variables were standardized (mean = 0, standard deviation = 1) before analysis; therefore estimates for models with exposure SUA denote the change in adipose tissue per one standard deviation of SUA. R^2 denotes the percentage of variance in the outcome that is explained by the model. P-values are

exploratory and not corrected for multiple testing. VAT: visceral adipose tissue; SAT: subcutaneous adipose tissue; HFF_HISTO: hepatic fat fraction as measured by high-speed T2-corrected multi-echo technique (HISTO); HFF_dixon: hepatic fat fraction, as measured by multiecho Dixon-sequence. Est.: Estimate

Supplementary Table S3: Sensitivity analysis: Association of SUA and Hyperuricemia with MRI-derived adipose tissue content adjusting for fasting serum glucose instead of diabetes

	VAT				SAT				HFF_HISTO				HFF_dixon			
	β	95%-CI	p-value	R^2	β	95%-CI	p-value	R^2	Estimate	95%-CI	p-value	R^2	Estimate	95%-CI	p-value	R^2
Exposure SUA																
fully adjusted	0.89	[0.64, 1.15]	2.73E-11	0.53	0.87	[0.45, 1.30]	5.92E-05	0.26	1.36	[1.25, 1.51]	5.17E-10	0.39	1.36	[1.23, 1.51]	1.28E-09	0.40
men	0.69	[0.38, 0.99]	1.83E-05	0.30	0.67	[0.27, 1.06]	0.001	0.25	1.21	[1.09, 1.35]	4.29E-04	0.26	1.20	[1.07, 1.32]	0.001	0.27
women	0.61	[0.36, 0.87]	5.15E-06	0.48	0.64	[0.02, 1.27]	0.042	0.21	1.36	[1.22, 1.54]	2.61E-07	0.42	1.39	[1.23, 1.57]	1.70E-07	0.44
fully adjusted + WC	0.41	[0.22, 0.60]	4.13E-05	0.75	-0.14	[-0.36, 0.09]	0.230	0.81	1.21	[1.11, 1.32]	3.05E-05	0.52	1.21	[1.11, 1.32]	6.33E-05	0.53
men	0.24	[0.02, 0.46]	0.034	0.66	-0.02	[-0.23, 0.19]	0.837	0.81	1.09	[0.99, 1.20]	0.074	0.44	1.08	[0.98, 1.20]	0.106	0.42
women	0.33	[0.15, 0.51]	5.29E-04	0.75	-0.19	[-0.50, 0.12]	0.227	0.81	1.27	[1.14, 1.42]	1.94E-05	0.51	1.28	[1.15, 1.42]	1.68E-05	0.56
fully adjusted + BMI	0.55	[0.33, 0.76]	6.25E-07	0.69	0.03	[-0.18, 0.24]	0.798	0.82	1.26	[1.15, 1.38]	1.30E-06	0.48	1.26	[1.14, 1.38]	2.90E-06	0.49
men	0.36	[0.11, 0.60]	0.005	0.57	0.09	[-0.12, 0.30]	0.405	0.80	1.12	[1.02, 1.23]	0.022	0.39	1.12	[1.01, 1.23]	0.035	0.37
women	0.39	[0.20, 0.58]	6.73E-05	0.73	-0.05	[-0.34, 0.24]	0.742	0.84	1.31	[1.17, 1.46]	4.78E-06	0.48	1.31	[1.17, 1.46]	3.90E-06	0.53
Exposure Hyperuricemia																

fully adjusted	1.58	[1.06, 2.09]	3.88E-09	0.52	1.2	[0.35, 2.05]	0.006	0.24	1.55	[1.27, 1.88]	1.80E-05	0.36	1.58	[1.30, 1.93]	9.59E-06	0.37
men	1.38	[0.71, 2.05]	6.89E-05	0.29	1.10	[0.23, 1.97]	0.014	0.24	1.43	[1.14, 1.80]	0.002	0.25	1.45	[1.15, 1.82]	0.002	0.27
women	1.81	[0.97, 2.65]	3.60E-05	0.46	1.47	[-0.56, 3.51]	0.154	0.19	1.80	[1.22, 2.69]	0.004	0.35	1.80	[1.20, 2.72]	0.005	0.36
fully adjusted + WC	0.89	[0.52, 1.27]	4.32E-06	0.75	-0.21	[-0.64, 0.23]	0.348	0.81	1.30	[1.08, 1.54]	4.75E-03	0.50	1.31	[1.09, 1.57]	0.003	0.52
men	0.63	[0.16, 1.10]	0.009	0.67	-0.06	[-0.51, 0.38]	0.776	0.81	1.20	[0.98, 1.46]	0.084	0.44	1.23	[1.00, 1.52]	0.049	0.42
women	1.22	[0.64, 1.80]	4.88E-05	0.75	-0.22	[-1.22, 0.78]	0.658	0.81	1.54	[1.07, 2.20]	0.020	0.46	1.48	[1.03, 2.14]	0.034	0.52
fully adjusted + BMI	1.12	[0.71, 1.53]	1.69E-07	0.70	0.11	[-0.30, 0.52]	0.602	0.82	1.38	[1.15, 1.65]	6.52E-04	0.46	1.40	[1.16, 1.70]	3.73E-04	0.47
men	0.92	[0.40, 1.44]	6.09E-04	0.58	0.3	[-0.16, 0.75]	0.202	0.80	1.28	[1.04, 1.58]	0.020	0.40	1.32	[1.06, 1.63]	0.012	0.38
women	1.20	[0.59, 1.80]	1.61E-04	0.73	-0.43	[-1.36, 0.50]	0.359	0.84	1.57	[1.07, 2.29]	0.020	0.42	1.51	[1.03, 2.20]	0.037	0.47

Results from a linear regression model with outcome adipose tissue. Exposure was either the continuous variable SUA or the dichotomous variable Hyperuricemia, defined as serum uric acid levels >6 mg/dL in women and >7 mg/dL in men. The fully adjusted model was adjusted for age, sex (if not sex-stratified), systolic blood pressure, total serum cholesterol, serum albumin, alcohol consumption, diuretic medication and fasting serum glucose. Continuous exposure and adjustment variables were standardized (mean = 0, standard deviation = 1) before analysis. Outcome hepatic fat fraction (HFF) was log-transformed and resulting estimates therefore denote the percent change in mean HFF. Continuous exposure and adjustment variables were standardized (mean = 0, standard deviation = 1) before analysis; therefore estimates for models with exposure SUA denote the change in adipose tissue per one standard deviation of SUA. R^2 denotes the percentage of variance in the outcome that is explained by the model. P-values are exploratory and not corrected for multiple testing. VAT: visceral adipose tissue; SAT: subcutaneous adipose tissue; HFF_HISTO: hepatic fat fraction as measured by high-speed T2-corrected multi-echo technique (HISTO); HFF_dixon: hepatic fat fraction, as measured by multiecho Dixon-sequence
